# Supplementary material for: Content-rich biological network constructed by mining PubMed abstracts
Source: BMC Bioinformatics. 2004 Oct 8;5:147. doi: 10.1186/1471-2105-5-147 (PMC528731; doi:10.1186/1471-2105-5-147)
Supplement: Additional File 2 — The original results of the above study (non-essential files are deleted to keep the file size under the limit set by BMC bioinformatics). [file 1471-2105-5-147-S2.bz2 › chilibotAdditionalFile2/dip05/691ID3056932E18012/html/HISJ_HISQ.html]

 


 **HISJ** and **HISQ** 
  
Found 12 abstracts in PubMed, retrieved 05.  
 

 What does Google say? 
 PDF only 
| .edu only 

---

**Interactive relationship** (e.g. stimulation, inhibition, etc)

**Non-interactive relationship** (e.g. studied together, co-existance, homology, etc.)

- In this in vitro preparation, transport activity is completely dependent on the presence of all four permease proteins  **HisJ** ,  **HisQ** , HisM, and HisP and on internal ATP.  Ref: 2674940 Proc Natl Acad Sci U S A, 1989
- The bacterial histidine permease, an ABC transporter, from Salmonella typhimurium is composed of a membrane bound complex, HisQMP2, comprising two hydrophobic subunits  **HisQ**  and HisM, two copies of an ATP hydrolyzing subunit, HisP, and a soluble receptor,  **HisJ** .  Ref: 11456221 J Bioenerg Biomembr, 2001
